# Digital Light Processing of ^19^F MRI-Traceable Gelatin-Based Biomaterial Inks towards Bone Tissue Regeneration

**DOI:** 10.3390/ma17122996

**Published:** 2024-06-19

**Authors:** Anna Szabó, Kristyna Kolouchova, Laurens Parmentier, Vit Herynek, Ondrej Groborz, Sandra Van Vlierberghe

**Affiliations:** 1 Polymer Chemistry and Biomaterials Group, Center of Macromolecular Chemistry, Department of Organic and Macromolecular Chemistry, Ghent University, Krijgslaan 281-S4, 9000 Ghent, Belgium; 2 Center for Advanced Preclinical Imaging (CAPI), First Faculty of Medicine, Charles University, Salmovská 3, 120 00 Prague, Czech Republic; 3 Institute of Organic Chemistry and Biochemistry, Czech Academy of Sciences, Flemingovo sq. 2, 160 00 Prague, Czech Republic; ondrej.groborz@seznam.cz; 4 Institute of Biophysics and Informatics, First Faculty of Medicine, Charles University, Salmovská 1, 120 00 Prague, Czech Republic; 5 BIO INX, Technologiepark-Zwijnaarde 66, 9052 Ghent, Belgium; 6 4Tissue, Technologiepark-Zwijnaarde 48, 9052 Ghent, Belgium

**Keywords:** tissue engineering, photo-crosslinkable gelatin, fluorine-19 magnetic resonance imaging, poly[*N*-(2,2-difluorethylacrylamide)], digital light processing

## Abstract

Gelatin-based photo-crosslinkable hydrogels are promising scaffold materials to serve regenerative medicine. They are widely applicable in additive manufacturing, which allows for the production of various scaffold microarchitectures in line with the anatomical requirements of the organ to be replaced or tissue defect to be treated. Upon their in vivo utilization, the main bottleneck is to monitor cell colonization along with their degradation (rate). In order to enable non-invasive visualization, labeling with MRI-active components like *N*-(2,2-difluoroethyl)acrylamide (DFEA) provides a promising approach. Herein, we report on the development of a gelatin-methacryloyl-aminoethyl-methacrylate-based biomaterial ink in combination with DFEA, applicable in digital light processing-based additive manufacturing towards bone tissue regeneration. The fabricated hydrogel constructs show excellent shape fidelity in line with the printing resolution, as DFEA acts as a small molecular crosslinker in the system. The constructs exhibit high stiffness (*E* = 36.9 ± 4.1 kPa, evaluated via oscillatory rheology), suitable to serve bone regeneration and excellent MRI visualization capacity. Moreover, in combination with adipose tissue-derived stem cells (ASCs), the 3D-printed constructs show biocompatibility, and upon 4 weeks of culture, the ASCs express the osteogenic differentiation marker Ca^2+^.

## 1. Introduction

Gelatin-based materials are one of the most studied hydrogels in regenerative medicine, since gelatin mimics the extracellular matrix (ECM) and supports cell adhesion and proliferation [1,2]. Additionally, gelatin has the capacity to be chemically modified, thereby allowing for tuning its physico-chemical and biological properties, or for introducing diagnostic modalities for future in vivo tracking capability, among others [3,4]. Moreover, gelatin can be chemically modified to introduce photo-crosslinkable moieties, e.g., methacrylamides (gelatin-methacryloyl, gel-MA) [5,6], to obtain shape fidelity upon applying UV–Vis irradiation in the presence of a suitable photo-initiator, thereby forming crosslinked hydrogel implants to serve regenerative applications [7]. To date, gel-MA is the gold standard gelatin derivative in the field of tissue engineering, although as a result of the relatively low achievable concentration of -MA moieties, extensive swelling of gel-MA-based structures often results in low spatial resolution upon light-based additive manufacturing [6]. To overcome this limitation, the further methacrylation of the carboxylic acid groups in gel-MA with 2-aminoethyl methacrylate (resulting in gelatin-methacryloyl-aminoethyl-methacrylate, gel-MA-AEMA) have offered a solution [6]. Via introducing a higher number of crosslinkable moieties on the gelatin backbone, a higher network density can be obtained following crosslinking, thereby resulting in limited swelling capacity and, therefore, outstanding spatial resolution. Moreover, gel-MA-AEMA has shown superior light-based additive manufacturing potential during development of medium- and high-resolution prints [8].

To further improve light-based processability, while tuning the physico-chemical and biological properties of gelatin-based hydrogels, supplementation of the formulations with various co-monomers offers a promising strategy [9]. For successful tissue regeneration, mimicking the physiological tissue properties upon scaffold development is a key requirement, as cells can mechanosense their microenvironment [10], which can affect their biological response [10,11]. Additionally, the biodegradation rate of implanted scaffolds should ideally match the rate of tissue regeneration. Monitoring the degradation of hydrogels in vivo is crucial, yet very challenging, which hinders their further entry into clinical applications [12,13].

Several studies reported on the implementation of various imageable hydrogels through fluorescence imaging [14], optical coherence tomography (OCT), ultrasound imaging (US) [15], X-ray imaging [16,17], PET [18], SPECT [19], MRI in the presence of contrast agents [20,21,22], or a chemical exchange saturation transfer (CEST) technique [20,21]. Although these methods can provide crude data about an implant’s biodegradation profile, these methods are usually associated with limited penetration depths (1 to 3 mm in OCT [23] and in vivo fluorescence [24]), expose the subject to ionizing radiation (X-ray, PET, SPECT), or are difficult to quantify (^1^H MRI, CEST MRI). Recently, we suggested that fluorine-19 magnetic resonance imaging (^19^F MRI) and spectroscopy (MRS) are promising for monitoring the degradation of implants in vivo both in a research setting and in clinical practice. Interestingly, ^19^F MRI/MRS has no limit regarding penetration depth, does not involve ionizing radiation, and is reasonably sensitive [25]. Limits of detection of ^19^F MRI are usually higher than a few milligrams per mL, which is ideal for larger scaffolds or tracer targeting and cumulation [25,26,27]. Moreover, ^19^F MRI can be executed with common ^1^H MRI scanners with only minor hardware adjustments (or can even be acquired simultaneously with ^1^H MRI scans) [28]. Previously, we incorporated *N*-(2,2-difluoroethyl)acrylamide (DFEA) into gelatin-based hydrogels [3,4], which endowed them with high amounts of magnetically equivalent fluorine atoms with optimal ^19^F MR properties [29] and thus enabled their ^19^F MRI/MRS detection [30,31,32]. Consequently, ^19^F MRI (combined with ^1^H MRI) scans can be used for anatomical localization of such implants, and ^19^F MRS can be used to quantify their biodegradation rates, as demonstrated in our previous research [3]. Nonetheless, despite the promising results of ^19^F MRI-detectable hydrogels, to date, such hydrogels have not yet been reported in combination with light-based additive manufacturing.

Light-based additive manufacturing (AM) techniques (including stereolithography, digital light processing, two-photon polymerization (2PP), etc.) enable superior computer-aided design (CAD)-based control of the obtained scaffold morphology and dimensions (down to the nanometer scale in the case of 2PP [33]) (compared to deposition-based AM) towards the development of advanced and patient-specific scaffolds exhibiting in vivo tissue mimicry [34,35]. Digital light processing (DLP) [36,37] is a time- and cost-efficient stereolithography-based technology, utilizing the projection of UV–Vis light on a digital micromirror device (DMD), transferred to an LED screen, to create complex, three-dimensional structures in a layer-by-layer fashion, enabling resolutions of 0.6–90 μm [38]. Upon the utilization of DLP-based AM, the development of a biomaterial ink formulation is required, encompassing a photo-crosslinkable polymer, a solvent, a photoinitiator (PI), and a photoabsorber (PA) in a well-balanced formulation to obtain high spatial resolution and excellent CAD/CAM mimicry [39].

**Figure 1:**
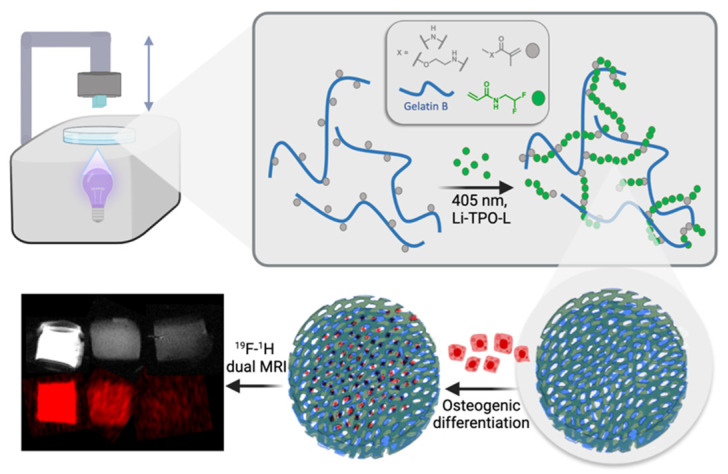
Schematic representation of the study’s approach: development of 3D-printable scaffolds incorporating gel-MA-AEMA, a photo-crosslinkable derivative of gelatin, and PDFEA, an ^19^F MRI tracer. The scaffolds are evaluated for their ability to support osteogenic differentiation, highlighting their potential in bone tissue engineering applications.

Herein, we explore the DLP-based AM processability of a novel gel-MA-AEMA-based biomaterial ink supplemented with DFEA towards the creation of ^19^F MRI-traceable scaffolds serving bone tissue engineering (Figure 1). Therefore, the current study reports on the optimization of the biomaterial ink composition to obtain mechanically stable (confirmed via oscillatory rheological measurements) porous scaffolds to be applied as cell carriers for osteogenic tissue regeneration. The ink composition is inspired based on our previously reported studies [3,4], yet the ideal composition utilizing gel-MA-AEMA and a specific DFEA content has been carefully selected to obtain scaffolds with ideal properties to support osteogenic differentiation. Subsequently, the scaffolds were combined with adipose tissue-derived stem cells (ASCs) to probe biocompatibility, cell adhesion, and their osteogenic differentiation potential. Finally, we demonstrated the ^19^F MRI detectability of these materials. Our results can be applied for printing of ^19^F MRI-detectable hydrogel implants serving regenerative medicine, whose biodegradation profile should be monitored in vivo.

## 2. Materials and Methods

### 2.1. Materials

2,2-Difluoroethylamine (97%) was purchased from Fluorochem (Derbyshire, UK). Triethylamine (TEA, ≥99%), acryloyl chloride (99%, ≈400 ppm phenothiazine), tartrazine (Acid Yellow 23), Dulbecco′s Modified Eagle′s Medium (DMEM), 10 *v*/*v*% fetal bovine serum, 2 *v*/*v*% calcein-acetoxymethyl (Ca-AM), Penicillin-Streptomycin, ascorbic acid, dexamethasone, β-glycerophosphate, propidium iodide (PI), dimethyl sulfoxide (DMSO-d6), and deuterium oxide were purchased from Sigma-Aldrich (Diegem, Belgium) and were used without any additional purifications. (2,4,6-trimethylbenzoyl)-phenyl-phosphinic acid ethyl ester was purchased from Speedcure TPO-L (Lambson, UK). 1-Ethyl-3-(3-dimethylamino)propyl)-carbodiimide hydrochloride (EDC) was obtained from TCI Europe (Zwijndrecht, Belgium). Dimethyl sulfoxide (DMSO, 99.85%) and *N*-hydroxysuccinimide (NHS, 98%) were purchased from Acros (Geel, Belgium). 2-Aminoethyl methacrylate hydrochloride (AEMA·HCl) was obtained from Polysciences (Conches, France). CaCl_2_ was acquired from Carl Roth (Brussel, Belgium). Deionized water used in all experiments (resistivity less than 18.2 MΩ·cm) was prepared using an Arium^®^ 611 (Sartorius, Goettingen, Germany) with the Sartopore 2 150 (0.45 + 0.2 µm pore size) cartridge filter. The adipose tissue-derived stem cells (ASCs) were purchased from Lonza (Basel, Switzerland). A Ca^2+^-assay kit was purchased from Sentinel diagnostics (Milano, Italy). *N*-(2,2-difluoroethyl)acrylamide (DFEA) was prepared as previously described through the reaction of 2,2-difluoroethylamine with acryloyl chloride in the presence of a triethylamine base [30].

### 2.2. Preparation of Gelatin-Methacryloyl-Aminoethyl-Methacrylate (Gel-MA-AEMA)

The preparation of the utilized gel-MA-AEMA was described in our previous study [8]. In brief, the first step involved the methacrylation of the amine functionalities in gelatin type B [40]. To this end, 100 g gelatin B was dissolved at 40 °C in 1.0 L phosphate buffer (pH 7.8). Subsequently, methacrylic anhydride (2.5 eq, 96.25 mmol, 14.34 mL) was added to the solution and stirred for 1 h. Thereafter, the product was dialyzed for 24 h at 40 °C against deionized water (DW, MWCO 12,000–14,000 Da), and the pH of the solution was adjusted to 7.3 followed by freeze-drying (freeze-dryer Alpha2, Martin Christ Gefriertrocknungsanlagen, Osterode am Harz, Germany). The second step of the functionalization involved the methacrylation of the intermediate gel-MA product onto the carboxylic acid moieties [6]. Therefore, 60 g gel-MA (66.0 mmol –COOH) was dissolved under inert atmosphere in 525 mL dry DMSO at 50 °C. Subsequently, EDC (0.50 eq.; 6.314 g; 0.033 mol) and 0.75 equivalents of NHS (5.687 g; 0.049 mol) were added to the solution in the presence 40 mL dry DMSO. After 30 min of activation time, 0.5 equivalents of 2-aminoethylmethacrylate hydrochloride (AEMA·HCl) (5.456 g; 0.033 mol) in the presence of 40 mL dry DMSO were added to the solution, followed by a 24 h reaction time, under inert atmosphere. Subsequently, the reaction mixture was precipitated in 10-fold acetone, filtered on a glass filter (number 4), and redissolved in double-distilled water (DDW). Thereafter, the product was dialyzed for 24 h at 40 °C against DW (MWCO 12,000–14,000 Da), and the pH of the solution was adjusted to 7.3, followed by freeze-drying (Christ freeze-dryer Alpha2).

### 2.3. Synthesis of Lithium Phenyl(2,4,6-Trimethylbenzoyl)phosphinate (Li-TPO-L)

Photoinitiator Li-TPO-L was prepared as previously described through the reaction of ethyl phenyl(2,4,6-trimethylbenzoyl)-phosphinate with lithium bromide in butan-2-one at 65 °C [33].

### 2.4. Evaluation of Photo-Crosslinkability of the Gel-MA-AEMA-F-Based Hydrogel Precursor via In Situ Photo-Rheology

The optimal ratio of gel-MA-AEMA co-polymerized with DFEA to obtain a good detectability by ^19^F MRI was previously reported by Kolouchova et al. [3,4] to be 1:20, and this was utilized in the current manuscript to prepare the hydrogel precursor solutions. To determine the crosslinkability and crosslinking kinetics of the hydrogel precursor solution, a Physica MCR-302 rheometer (Anton Paar, Graz, Austria) was utilized with a parallel plate setup (upper plate diameter *d* = 25 mm). To this end, 300 µL of the gel-MA-AEMA-DFEA hydrogel precursor mixture (gel-MA-AEMA-F throughout the manuscript, consisting of 150 mg gel-MA-AEMA, 97.0 μmol of MA+AEMA moieties, 260 mg DFEA (1.94 mmol), 1 mL dry DMSO, in the presence of 2 mol% Li-TPO-L with regard to the molar amount of photo-crosslinkable moieties, bubbled with argon for 30 s) was pipetted to the bottom plate of the rheometer. As negative control samples, gel-MA-AEMA films without the supplementation of DFEA were prepared under the same conditions. The samples were irradiated with UV-A light (320 to 500 nm, OmniCure 1500 UV light source (Efsen UV & EB Technology, Holte, Denmark), light intensity *I* = 10 mW/cm^2^) at 37 °C. The frequency was set to 1 Hz, at a strain of 0.1% and *F*_N_ = 0.6 N, to evaluate the storage (*G*′) and loss moduli (*G*″) as a function of time.

### 2.5. Mechanical Evaluation of the Gel-MA-AEMA-F Hydrogels via Oscillatory Rheology

The gel-MA-AEMA-F solution was prepared as described in Section 2.5. As negative control samples, gel-MA-AEMA films without the supplementation of DFEA were prepared under the same conditions. In brief, the gel-MA-AEMA-F solution was transferred between two parallel glass plates covered with PP foil and separated with a silicone spacer (thickness 1 mm). Film casting was followed by 30 min UV-A irradiation from both sides at *I* = 10 mW/cm^2^, λ = 365 nm. Subsequent to film casting, the samples were washed in 70% EtOH to remove possibly residual non-crosslinked DFEA moieties or gel-MA-AEMA and swollen in DDW for 24 h at 37 °C.

To evaluate the mechanical properties of the equilibrium-swollen hydrogels, 14 mm diameter discs were punched out and transferred to the bottom plate of a Physica MCR-301 rheometer with parallel plate setup (upper plate diameter *d* = 15 mm). During the measurement, 0.1% strain and a normal force *F*_N_ of 0.6 N were applied on the samples to study the storage modulus (*G*′) as a function of frequency (measured range from 0.1 to 10 Hz). The storage (*G*′) modulus was evaluated at a frequency of 1 Hz. To gain elastic modulus values, the formula *E* = 3·*G*′ [41] was utilized.

### 2.6. Biomaterial Ink Development

#### 2.6.1. Creation of a Computer-Aided Design (CAD)

**Figure 2:**
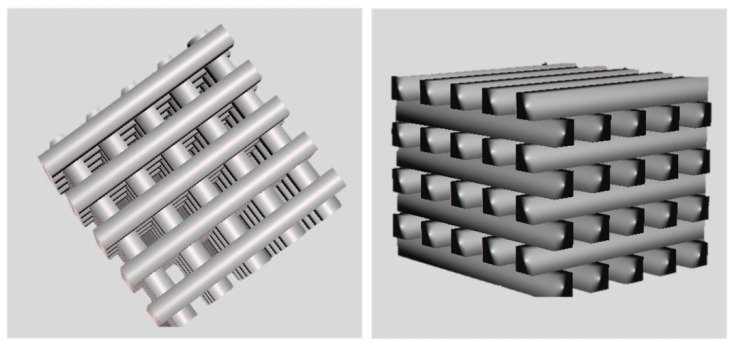
CAD design of 3D-porous scaffolds. The dimensions of the scaffold are 5 mm × 5 mm × 4 mm, with a size of 500 µm for pores and struts.

SolidWorks 2021 was utilized to acquire a 3D CAD to favor well-vascularized scaffolds exhibiting direct osteogenesis in vivo [42,43]. To this end, a porous design was created with geometrical quantities (pore sizes larger than 300 µm, in line with the state of the art, namely, 500 µm, accompanied by 500 µm struts in diameter), as shown in Figure 2.

#### 2.6.2. In Situ Rheological Evaluation of the Crosslinking Kinetics of Different Biomaterial Ink Formulations

Gel-MA-AEMA (150 mg; 97 μmol of MA + AEMA moieties) was dissolved in the presence of DFEA monomer (260 mg, 1.94 mmol) in dry DMSO (1.0 mL) at 50 °C, followed by bubbling with argon for 30 s. Thereafter, different concentrations (mol%) of Li-TPO-L (10 to 20 mol%) as a photo-initiator (PI) and tartrazine as a photo-absorber (PA, 0.5 to 0.8 mol%) with respect to the amount of photo-crosslinkable moieties present in the hydrogel precursors were added to the system and evaluated via in situ rheological measurements to assess the crosslinking kinetics and gel point (*t*_gel_, the cross-over point of *G*′ and *G*″) [8,44]. To this end, a Physica MCR-302 rheometer (Anton Paar, Sint-Martens-Latem, Belgium) with parallel plate setup was utilized, coupled with an OmniCure 1500 UV light source (*λ* = 400 to 500 nm). A total of 300 µL of the biomaterial ink composition was transferred to the bottom plate of the rheometer and irradiated at *I* = 19.51 mW/cm^2^ at 37 °C while applying *f* = 1 Hz, *γ* = 0.1%, and *F*_N_ = 0.5 N, *n* = 3. The storage (*G*′) and loss moduli (*G*″) were monitored as a function of time to assess *t*_gel_.

#### 2.6.3. DLP Processing of the Gel-MA-AEMA-F Biomaterial Ink

The final composition of the gel-MA-AEMA-F consisted of 150 mg gel-MA-AEMA, 260 mg DFEA in the presence of 20 mol% Li-TPO-L, and 0.72 mol% tartrazine with respect to the double bonds present in the precursors dissolved in 1 mL of dry DMSO. The solution was transferred to the vat of the printer (CellInk LumenX, Gothenburg, Sweden). The printing parameters were 19.51 mW/cm^2^ intensity at *λ* = 405 nm, 2.5 s curing time/layer at room temperature. Subsequently, the scaffolds were washed for 24 h (EtOH:water 70:30) to remove unreacted species and equilibrium-swollen in PBS (140 mM pH 7.4) for 24 h at 37 °C.

### 2.7. Morphological Evaluation of the 3D Hydrogel Constructs

The equilibrium-swollen hydrogel constructs were observed via optical and scanning electron microscopy (SEM). To this end, a Zeiss Axiotech 100 HD/DIC optical microscope was exploited (Carl Zeiss AG, Oberkochen, Germany) at 5× magnification.

Subsequently, the samples were visualized via SEM (JCM-7000 NeoScope™ Benchtop SEM, JEOL Europe BV, Nieuw-Vennep, The Netherlands). The samples for SEM were equilibrium-swollen in DDW to avoid salt crystallization upon drying, followed by vacuum drying, and coated with a nano-layer of gold using an Automatic Sputter Coater K550X with an RV3 two-stage rotary vane pump (Emitech/Quorum Tech., East Sussex, UK).

### 2.8. High-Resolution Magic Angle Spinning (HR-MAS) ^1^H-NMR Spectroscopy

To determine the crosslinking efficiency, the 3D hydrogel constructs were freeze-dried, reswollen in DMSO-d6, and measured with a Bruker WH 500 MHz (Bruker, Billerica, MA, USA) to perform high-resolution magic angle spinning (HR-MAS) ^1^H-NMR spectroscopy. The HR-MAS spectrum was recorded at a spinning frequency of 6000 Hz, at room temperature (25 °C), and processed with MestReNova software, v. 12.0.3. The crosslinking efficiency was determined through Equation (1), where *DS_C_* reflects the degree of substitution of the crosslinked hydrogel, and *DS_UC_* is the *DS* of the uncrosslinked hydrogel.

(1) $$Crosslinking\;\mathrm{efficiency}=\frac{{DS}_{UC}-{DS}_{C}}{{DS}_{UC}}\cdot 100\%$$

### 2.9. Determination of Swelling Ratio (SR) and Gel Fraction (GF)

To determine the swelling ratio and gel fraction, the samples were freeze-dried immediately after crosslinking, followed by the evaluation of the sample mass (*m_d_*_,1_). Then, the scaffolds were incubated in DDW at 37 °C for 24 h to obtain equilibrium swelling. Thereafter, the swollen mass of the samples was determined (*m_s_*), followed by another freeze-drying step, to be able to determine the second dry mass of the scaffolds (*m_d_*_,2_). The gel fraction was determined via Equation (2), and the equilibrium swelling ratio (*SR*) was calculated using Equation (3).

(2) $$GF\;\left(\%\right)=\frac{m_{d,1}}{m_{d,2}}\;\cdot 100\%$$

(3) $$SR=\frac{m_{s}}{m_{d}}\;$$

All measurements were performed in triplicate. As negative control samples, gel-MA-AEMA films without the supplementation of DFEA were prepared under the same conditions.

### 2.10. Elemental Analysis of the DLP-Processed Gel-MA-AEMA-F Scaffolds

The C/H/N composition of freeze-dried hydrogels was measured using a PE 2400 Series II CHNS/O analyzer (Perkin Elmer, Waltham, MA, USA) in combustion mode (pure oxygen). Ash of all samples was 1.5 ± 0.1 mg (all samples were weighted with calibrated scale with accuracy of 0.001 mg). The absolute uncertainty of CHN determination is ≤0.3 abs. %. The instrument was calibrated before use. All samples were measured in 2 independent experiments. For assessment of fluorine content, freeze-dried samples were combusted in an atmosphere of pure oxygen. Next, the sample residue was washed with pure water, and the fluoride content in this solution was assessed with a fluoride-selective electrode (Orion Star, A214, Thermo Scientific; Waltham, MA, USA).

### 2.11. Acquisition of ^19^F Magnetic Resonance Spectroscopy (^19^F MRS) and Imaging (^19^F MRI)

A set of phantoms bases on 3D-printed scaffolds was prepared. The 3D-printed scaffolds (76.6 mg) were cut into smaller parts with a mass of 38.3, 19.1, 9.5, and 4.7 mg. They were positioned in a 12-well plate and underwent ^19^F MRS and MRI measurements. The phantoms were measured at room temperature using an animal 7 T MRI scanner (MR Solutions, Guildford, UK) equipped with a ^1^H/^19^F dual whole-body volume coil for rats. ^19^F MR spectra were acquired by a one-pulse FID sequence (bandwidth 50 kHz, number of acquisitions *NA* = 64). Subsequently, we acquired ^1^H MR images using a T1-weighted turbospin echo sequence (echo time *TE* = 8 ms, turbofactor *TF* = 4, repetition time *TR* = 1000 ms, number of acquisitions *NA* = 16, matrix 128 × 128, field of view *FOV* = 20 × 40 mm^2^, slice thickness 1.0 mm) and ^19^F MR images using a modified turbospin echo sequence (*TE* = 8 ms, *TF* = 8, *TR* = 1000 ms, *NA* = 512, matrix 64 × 64, *FOV* = 20 × 40 mm^2^, slice thickness 10 mm). Finally, we interpolated ^19^F images to the same image matrix as was acquired in the case of ^1^H MRI, then color-coded (red) and merged these with ^1^H images (grayscale) using ImageJ software, v. 1.53t [45].

### 2.12. Biological Evaluation of the Gel-MA-AEMA-F Hydrogel Scaffolds

In vitro cell assays were performed in line with ISO-10993 to evaluate the biocompatibility through cell viability quantification and morphology and the cell colonization assessment of seeded adipose tissue-derived stem cells (ASCs) onto the 3D-printed scaffolds. Moreover, the induction of stem cell osteogenic differentiation was evaluated through a Ca^2+^-assay as a late osteogenic marker.

#### 2.12.1. Cell Culture Conditions

Three-dimensional-printed scaffolds were sterilized through a 12 h incubation in 70 *v*/*v*% ethanol solution followed by 2 h of UV-C irradiation (100 to 280 nm, 15 mW/cm^2^). The sterilized samples were then swollen again at 37 °C in Dulbecco’s Modified Eagle Medium (DMEM), supplemented with 10 *v*/*v*% fetal bovine serum (FBS) and 1 *v*/*v*% penicillin/streptomycin. The adipose tissue-derived stem cells (ASCs) were cultured in the same medium under standard incubator conditions (37 °C, 5% CO_2_) and with a culture medium change occurring twice a week. ASCs at passage 4 were carefully seeded at a density of 100,000 ASCs/cm^2^ on the sterilized 3D-printed scaffolds. At day 3, the attachment of the cells to the scaffolds was checked with the microscope, and the samples were transferred to new well plates containing osteogenic medium (culture medium supplemented with 0.08 mM ascorbic acid, 100 nM dexamethasone, and 10 mM β-glycerophosphate) in order to separate the cells interacting with the scaffolds from the cells growing on the bottom of the well plate.

#### 2.12.2. Evaluation of Biocompatibility of the Gel-MA-AEMA-F Hydrogel Constructs via Live/Dead Staining

At four consecutive time points (days 7, 14, 21 and 28), the biocompatibility—as reflected by the cell viability and morphology—was assessed through live/dead staining. After discarding the culture medium from the cell-seeded constructs, 2 *v*/*v*% calcein-acetoxymethyl (Ca-AM)/propidium iodide (PI) staining solution in PBS was added. After incubation of the samples in the dark for 10 min, the ASCs were visualized through a green fluorescent protein (GFP) and a Texas Red (TxRed) filter of a confocal scanning microscope (LSM710, Carl Zeiss AG, Oberkochen, Germany). The percentage viability was quantified using FIJI software, 2.14.0/1.54f.

#### 2.12.3. Evaluation of ASC Osteogenic Differentiation Capacity via a Ca^2+^-Assay

Additionally, at day 28, three ASC-seeded scaffolds were digested overnight in 1 M HCl at 60 °C. A 5-point triplicate calibration curve was made through serial dilutions of 1 M CaCl_2_. Standards and samples (10 µL) were then combined with 140 µL of the cresolphtalein-based working dye solution, followed by incubation in the dark at room temperature for 10 min. Thereafter, the absorbance was measured at 580 nm (and 750 nm for background correction) with a spectrophotometer (Tecan Infinite M200 Pro, Tecan, Männedorf, Switzerland).

### 2.13. Statistical Analysis

At least 3 replicates were exploited in each experiment, and GraphPad Prism 8 was utilized to perform one-way variation analysis (ANOVA) and student’s *t*-tests, unless otherwise stated, on the maintained mean ± standard values. Results were considered statistically significant in the case of *p* < 0.05.

## 3. Results and Discussion

### 3.1. Development of Hydrogel Precursors

The development and characterization of *N*-(2,2-difluoroethyl)acrylamide (DFEA) and gelatin-methacryloyl-aminoethyl-methacrylate was previously described in detail by our group [30].

### 3.2. Physico-Chemical Evaluation of the Gel-MA-AEMA-F Hydrogels

In order to determine the mechanical properties of the crosslinked, equilibrium-swollen hydrogels, oscillatory rheology was applied. The gel-MA-AEMA-F hydrogel films exhibited a *G*′ of 12.3 ± 1.4 kPa, corresponding to *E*′ = 36.9 ± 4.1 kPa, calculated based on description in Chapter 2.5 (Table 1, Figure S2). As a negative control, gel-MA-AEMA hydrogel films were also studied, lacking the DFEA component. They showed a *G*′ = 5.1 ± 2.1 kPa corresponding to *E*′ = 15.4 ± 6.2 kPa (Figure S1), which implies a significant strengthening effect (*p* = 0.0078) resulting from DFEA on the mechanical properties of gel-MA-AEMA, as a result of the higher crosslinking density [46]. The obtained results are in agreement with the mechanical requirements of bone osteoid extracellular matrix tissue (*E*′ ≈ 25–40 kPa) [47]. Therefore, the hydrogels were evaluated for their potential to support osteogenic differentiation of adipose tissue-derived stem cells (ASCs). Moreover, the mechanical properties and swelling of such gelatin-based materials can be largely tuned by changing the concentration of the starting materials [6]. Therefore, such materials can be fine-tuned to serve various applications in regenerative medicine.

Moreover, the swelling degree and gel fraction of the developed gel-MA-AEMA-based hydrogel hydrogels were evaluated. Firstly, the gel fraction of gel-MA-AEMA films without the presence of DFEA was previously assessed (Table 1). Upon crosslinking gel-MA-AEMA, a gel fraction of 97.1 ± 0.1% was achieved, which implies efficient crosslinking of the hydrogel precursor, in line with previous reports of Szabó et al. (*GF* = 98.2 ± 2.5%) [48]. They previously reported on the swelling degree of crosslinked gel-MA-AEMA 15 *w*/*v*% in PBS, being 12.1 ± 0.7 for 2D films, which is in line with the present work (Table 1, *SR* = 11.9 ± 0.1, Table 1).

Upon supplementing the gel-MA-AEMA precursors with DFEA, the gel fraction of the 2D-crosslinked films decreases to 73.0 ± 1.12, due to the washing out of non-reacted DFEA monomer, which is in line with previous studies [3,4]. The swelling ratio of the gel-MA-AEMA-F hydrogels was 4.8 ± 0.37 for 2D film, which suggests a significant decrease in the swelling capacity of the gel-MA-AEMA component upon supplementing it with a small crosslinker molecule, herein DFEA (*p* < 0.0001). The results are in line with our previous study, where the addition of DFEA significantly decreases the swelling ratio of the modified gelatins [3,4]. The reason for this decrease in the *SR* is due to the hydrophobic nature of the PDFEA linkers and offers the potential to provide superior CAD/CAM mimicry to the hereby developed gel-MA-AEMA-F biomaterial inks.

### 3.3. DLP Processability of the Biomaterial Ink towards the Fabrication of a Microporous 3D Architecture

The optimization of the final biomaterial ink composition, with regard to the photoinitiator and photoabsorber content, was performed by in situ rheology to assess the gelation time of the biomaterial ink as a function of PI and PA content (Table S1, Figures S3–S7).

**Figure 3:**
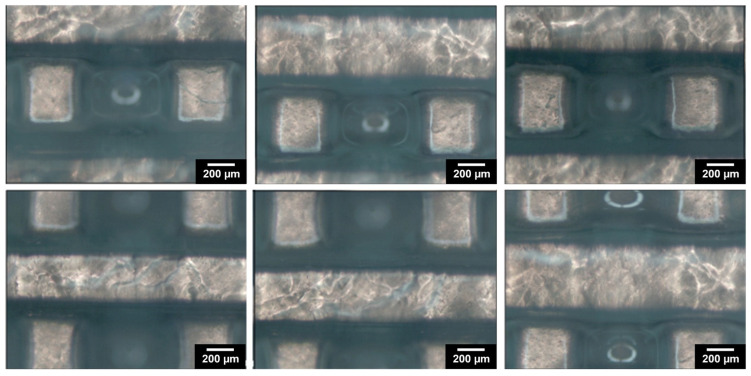
The morphology of the designed 3D-porous scaffold: optical microscopy images of washed 3D-printed scaffold, reswollen in PBS at 37 °C.

**Figure 4:**
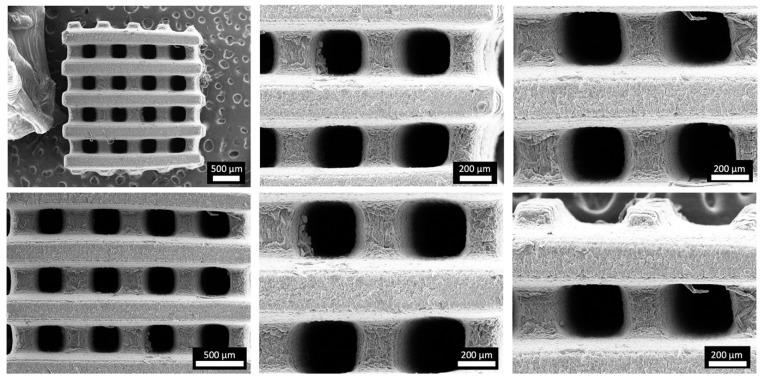
Morphology of the 3D-printed porous scaffold: SEM micrographs of vacuum-dried 3D-printed scaffold at different magnifications.

Figure 1 depicts the computer-aided design consisting of 500 µm struts in diameter with 5 mm strut length and with a total scaffold height of 4 mm. Figure 3 depicts optical microscopy images of the equilibrium-swollen (in PBS) hydrogel scaffolds. The obtained strut diameter after equilibrium swelling in PBS corresponds to 369 ± 55 µm, which implies a CAD/CAM mimicry of 74 ± 11%, and the pore size corresponds to 582 ± 43 µm, implying a 116 ± 9% CAD/CAM mimicry. The major changes in strut and pore size compared to the original CAD design originate from the solvent exchange (from DMSO to PBS). As DMSO dissolves DFEA, poly(DFEA), and gel-MA-AEMA, whereas PBS is only an efficient solvent for gel-MA-AEMA, the struts shrink during solvent exchange (hence the low swelling capacity, see Section 3.4.2) [4]. Szabó et al. [48] previously reported on the spatial CAD/CAM mimicry for a gel-MA-AEMA-based biomaterial ink composition, being 101 ± 6%, although in that work, processing of the hydrogel constructs was performed in PBS to avoid major changes in the final construct morphology [8,48]. Figure 4 depicts SEM micrographs of dry 3D-porous scaffolds.

Overall, the utilization of DFEA as an MRI-traceable co-monomer does not only provide the diagnostic function to the scaffold in a non-invasive manner but offers morphological integrity and shape fidelity of the 3D-processed hydrogel constructs towards reinforced scaffolds serving hard tissue engineering purposes.

### 3.4. Physico-Chemical Evaluation of the 3D Hydrogel Constructs

#### 3.4.1. Evaluation of the Composition and Crosslinking Efficiency of the 3D-Processed Hydrogel Precursors

The results from elemental analysis confirmed that only a portion of DFEA monomer from the reaction mixture was incorporated into the final materials (75% from the initial amount, Table 1), which is in line with our previous study [4]. The DFEA content in the final hydrogels after washing was calculated from the wt. % of F, which constitutes 28.12 wt. % of the molar mass of DFEA monomer.

The crosslinking efficiency (double-bond conversion) assessed from HR-MAS NMR data of the 3D hydrogel constructs was shown to be 99% regarding both the -MA and -AEMA moieties in gel-MA-AEMA (Figure S8). To this end, we can conclude that the constructs exhibited almost complete double-bond conversion of the gel-MA-AEMA component, although as shown in the elemental analysis, 25% of the DFEA remained uncrosslinked and leached out during the washing step, which is in line with our previous study [4]. Szabó et al. previously reported on the crosslinking efficiency of gel-MA-AEMA in the context of DLP processing, which resulted in 100% crosslinking efficiency regarding the -MA moieties and 96% for the -AEMA functionalities. Therefore, our findings are in line with previous reports [3,4,48].

#### 3.4.2. Swelling Ratio and Gel Fraction of 3D-Processed Hydrogel Precursors

Next, the swelling ratio and gel fraction of 3D gel-MA-AEMA-F constructs were evaluated (Table 1). DLP-fabricated 3D constructs typically require post-curing to enable sufficient crosslinking [49]. However, as confirmed by HR-MAS, due to the excellent crosslinking efficiency, no post-curing was required herein. However, the gel fraction value obtained for gel-MA-AEMA-F was 48.5 ± 1.1%, which implies major leaching out of formulation constituents. This is in line with the results of the elemental analysis, which suggested that 25% of the DFEA remained unreacted during DLP processing and leached out.

#### 3.4.3. ^19^F MRI Detectability of 3D Hydrogel Precursors

**Figure 5:**
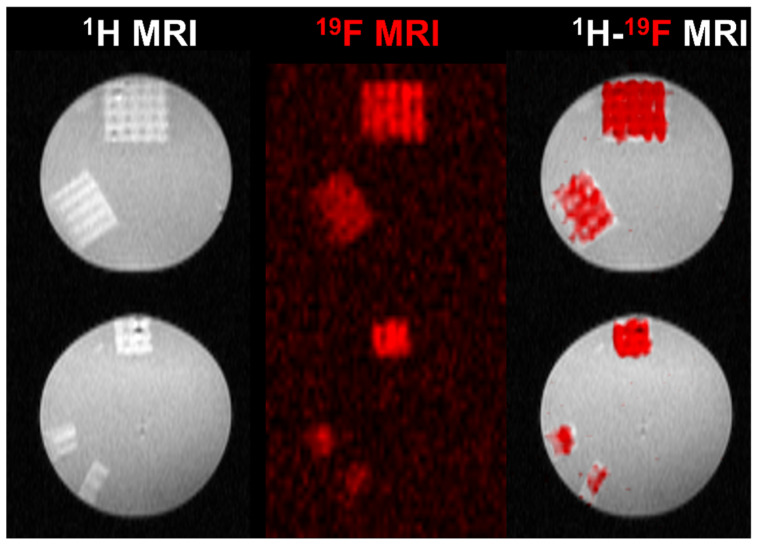
^1^H (gray scale), ^19^F (red scale), and dual ^1^H-^19^F MRI of prepared 3D-printed scaffolds reswollen in PBS (140 mM, pH 7.4) measured at 30 °C. The scaffold mass was (top to bottom) 76.6, 38.3, 19.1, 9.5, and 4.7 mg.

The PDFEA-based polymers have a high content of magnetically equivalent fluorine atoms with suitable properties for ^19^F MRI visualization [30,31,32]. We assessed the detection limits of the 3D-printed scaffolds using ^19^F MRI in vitro to determine the minimum size of the scaffold, which can be still detected in vivo (i.e., within a 20 min acquisition time). The 3D-printed scaffold (76.6 mg) was measured and then divided into smaller pieces with masses of 38.3, 19.1, 9.5, and 4.7 mg (Figure 5). All scaffolds could be visualized using ^1^H and ^19^F MRI. The limit of detection was well below 4.7 mg of hydrogel (1.9 mg of dry material, corresponding to approximately 0.25 mg of fluorine). The signal-to-noise ratio (SNR) varied from 4 (the smallest sample) to 7. Rather low SNR in the case of larger samples reflected the heterogeneous ^19^F signal within each sample caused by the structure of the 3D print (minimum signal was 7 a.u., maximum 55, average signal was 30 a.u. in the first sample). In comparison, the PDFEA-based polymers can usually be detected at concentrations above 3 mg/mL [30,50]. Such low detection limits ensure a good detectability of depots even in later phases of degradation and may enable the monitoring of hydrogel degradation at the site of implantation. These results demonstrate the great potential of the synthetized materials in future medical practice, especially in applications where materials are used in rather large volumes (e.g., regenerative medicine).

### 3.5. Biological Evaluation of the 3D Gel-MA-AEMA-F Hydrogel Constructs

**Figure 6:**
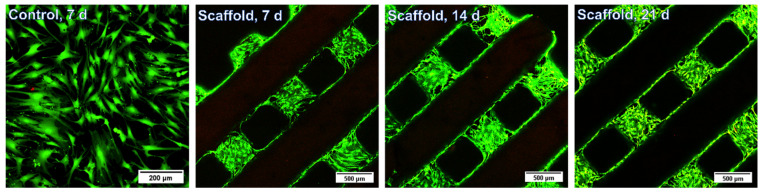
In vitro live/dead staining of seeded adipose tissue-derived stem cells after 7 d, 14 d, and 21 d. Scale bars represent 200 µm for the control and 500 µm for the scaffolds.

The suitability of the gel-MA-AEMA towards tissue engineering has been previously described [6,48]. However, the addition of a co-crosslinker can hamper the cell interactivity as well as the biocompatibility of the resulting materials [51,52]. Therefore, we assessed the suitability of the gel-MA-AEMA-F materials towards tissue engineering in a direct cell-seeding study. As was discussed in Section 3.2, the *E*′ of the developed gel-MA-AEMA-F hydrogels is 36.9 ± 4.1 kPa, which falls in the range of the mechanical requirements of bone osteoid extracellular matrix tissue (*E*′ = 25–40 kPa), as Engler et al. suggest [47]. Therefore, we assessed the suitability of the developed materials for adipose tissue-derived cell culture and their differentiation into osteogenic cells during a 1-month in vitro cell-seeding study. The materials were non-cytotoxic (viabilities > 96%, in line with the previous study of Van Hoorick et al. [6]) (Figure S9), and the seeded ASCs colonized the 3D-printed scaffold over time (Figure 6 and Figures S10–S16). Moreover, after 28 days, the seeded cells produced calcium deposits (3.2 ± 0.8 ng Ca^2+^/scaffold) as late osteogenic differentiation markers, hereby underlining the suitability of the scaffolds to serve bone tissue engineering.

## 4. Conclusions

Herein, we demonstrated the additive manufacturing capability of a gelatin-methacryloyl-aminoethyl-methacrylate and *N*-(2,2-difluoroethyl)acrylamide-based biomaterial ink to support bone regeneration. The hydrogel constructs showed a CAD/CAM mimicry of 74 ± 11% and allowed for the formation of 582 ± 43 µm wide pores, which are required to support osteogenic differentiation. The biomaterial ink showed appropriate stiffness (*E*′ = 36.9 ± 4.1 kPa, evaluated via oscillatory rheology), in line with the physiological osteoid extracellular matrix stiffness (25–40 kPa) [47]. Moreover, as a result of the incorporation of DFEA as crosslinker in the biomaterial ink composition, the constructs showed outstanding MRI visualization capability (detection limits below 0.25 mg of fluorine, corresponding to 4.7 mg of the swollen hydrogel), providing a possibility for long- term non-invasive monitoring of the scaffolds upon in vivo implantation. Finally, the constructs were successfully combined with adipose tissue-derived stem cells (ASCs), evidencing biocompatibility (viabilities > 96%) and osteogenic differentiation of the ASCs following 4 weeks of culture, as demonstrated via a Ca^2+^-assay.

In conclusion, this work represents a proof-of-concept application of an MRI-traceable biomaterial ink towards the non-invasive biomedical imaging of implants. Thanks to the diversity of the computer-aided design (CAD), 3D additive manufacturing offers outstanding design possibilities regarding complexity towards in vivo mimicry of human tissues. Moreover, as DLP is a time- and cost-efficient technique, rapid prototyping of the scaffolds can be foreseen in a timely manner, while offering sufficient resolution, in line with the equipment capabilities. However, as currently available DLP 3D printers utilized for biomedical applications are limited, to date, high-throughput commercial utilization of the technique is yet to come. Forthcoming research will focus on the potential variability of MRI signal intensity in line with different applications, after which potential upscaling of the biomaterial ink for commercial purposes can be considered.

## Figures and Tables

**Table 1:** Characteristics of resulting crosslinked gelatin-PDFEA hydrogels: composition and determined gel fraction and swelling ratios.

Material	*c*_gel_ (*w*/*v*%)	^19^F ± SD ^a^* (wt. %)	*n*_DFEA_/*n*_MA+AEMA_ Hydrogels *	*SR* ± SD *	*GF* ± SD * (%)	*G*′ (kPa) ^#^	*E*′ (kPa)
Gel-MA-AEMA-F ^b^	15.0	13.1	15	2.5 ± 0.02	48.5 ± 1.1	12.3 ± 1.4	36.9 ± 4.1
Gel-MA-AEMA ^c^	15.0	0.0	0	11.9 ± 0.1 ^#^	97.1 ± 0.1 ^#^	5.1 ± 2.1	15.4 ± 6.2

^a^ Determined by elemental analysis for dry material. ^b^ Determined subsequent to DLP processing. ^c^ Determined subsequent to UV-crosslinking [48]. * SD calculated for n = 3. ^#^ Evaluated from films.

## Data Availability

Raw data are available upon reasonable request.

## Supplementary Materials

The following supporting information can be downloaded at: https://www.mdpi.com/article/10.3390/ma17122996/s1, Figure S1: Mechanical spectrum of the hydrogel gel-MA AEMA (15 *w*/*v* %, 2 mol. % of PI resp. to MA/AEMA content). The storage (G’) and loss (G’’) moduli were measured at 37 °C, normal force 0.6 N, and strain of 0.1%. The experiment was measured in three runs; Figure S2: Mechanical spectrum of the hydrogel gel-MA AEMA-F (2 mol. % of PI resp. to MA/AEMA content). The storage (G’) and loss (G’’) moduli were measured at 37 °C, normal force 0.6 N, and strain of 0.1%. The experiment was measured in three runs; Table S1: The effect of content of Li-TPO-L (photo-initiator) and tartrazine (photo-absorber) in the starting solution for DLP optimization on its tg measured by rheology; Figure S3: Evolution of the storage modulus of 15 wt. % gel-MA-AEMA-F in DMSO with the 10 mol. % concentration of PI, respectively to molar amount of the MA/AEMA, during UV-A-induced cross-linking at 37 °C as determined by rheology. The experiment was measured in three runs; Figure S4: Evolution of the storage modulus of 15 wt. % gel-MA-AEMA-F in DMSO with the 15 mol. % concentration of PI, respectively to molar amount of the MA/AEMA, during UV-A-induced cross-linking at 37 °C as determined by rheology. The experiment was measured in three runs; Figure S5: Evolution of the storage modulus of 15 wt. % gel-MA-AEMA-F in DMSO with the 20 mol. % concentration of PI, respectively to molar amount of the MA/AEMA, during UV-A-induced cross-linking at 37 °C as determined by rheology. The experiment was measured in three runs; Figure S6: Evolution of the storage modulus of 15 wt. % gel-MA-AEMA-F in DMSO with the 20 mol. % concentration of PI and 0.5 mol. % concentration of PA, respectively to molar amount of the MA/AEMA, during UV-A-induced cross-linking at 37 °C as determined by rheology. The experiment was measured in three runs; Figure S7: Evolution of the storage modulus of 15 wt. % gel-MA-AEMA-F in DMSO with the 20 mol. % concentration of PI and 0.8 mol. % concentration of PA, respectively to molar amount of the MA/AEMA, during UV-A-induced cross-linking at 37 °C as determined by rheology. The experiment was measured in three runs; Figure S8: 1H HR MAS NMR of a sample gel-MA-AEMA-F swollen in DMSO-d6 measured using Bruker Avance 400 MHz spectrometer (9.4 T wide bore magnet) equipped with a 4 mm BL4 X/Y/H probe. Magic angle spinning was performed at 4 kHz using ceramic zirconia rotors of 4 mm in diameter. Acquisition parameters used were the following: a spectral width of 8 kHz, a 90° pulse length of 3.5 μs, an acquisition time of 1.7 s, a recycle delay time of 15 s and about 64 accumulations; Figure S9: Evaluation of in vitro live/dead assay of seeded adipose tissue-derived stem cells onto scaffolds after 7, 14, and 21 d compared to the control (osteogenic tissue-derived stem cells seeded on a well plate), evaluated after 7 d; Figure S10: In vitro live/dead staining of seeded adipose tissue-derived stem cells after 7 d (1 selected scaffold from 3). Series of stack images (2 µm thick) of populated scaffold; Figure S11: In vitro live/dead staining of seeded adipose tissue-derived stem cells after 14 d (1 selected scaffold from 3). Series of stack images (2 µm thick) of populated scaffold; Figure S12: In vitro live/dead staining of seeded adipose tissue-derived stem cells after 21 d (1 selected scaffold from 3). Series of stack images (2 µm thick) of populated scaffold; Figure S13: In vitro live/dead staining of 3 control samples, the adipose tissue-derived stem cells seeded on a well plate, after 7 d; Figure S14: In vitro live/dead staining of adipose tissue-derived stem cells seeded on 3 printed scaffolds after 7 d; Figure S15: In vitro live/dead staining of adipose tissue-derived stem cells seeded on 3 printed scaffolds after 14 d; Figure S16: In vitro live/dead staining of adipose tissue-derived stem cells seeded on 3 printed scaffolds after 21 d. References [53,54,55,56] are cited in the Supplementary Materials.
