# Supplementary material for: Digital Light Processing of 19F MRI-Traceable Gelatin-Based Biomaterial Inks towards Bone Tissue Regeneration
Source: Materials (Basel). 2024 Jun 19;17(12):2996. doi: 10.3390/ma17122996 (PMC11206011; doi:10.3390/ma17122996)
Supplement: Supplementary file 1 [file materials-17-02996-s001.zip › materials-2946754-supplementary.pdf]

Supporting information to the article

Digital light processing of ^{19}F MRI traceable gelatin-based biomaterial inks towards bone tissue regeneration

Anna Szabó,[1] Kristyna Kolouchova,[1] Laurens Parmentier,[1] Vit Herynek,[2] Ondrej Groborz,[3,4] Sandra Van Vlierberghe[1,5,6*]*

[1]Polymer Chemistry and Biomaterials Group, Centre of Macromolecular Chemistry, Department of Organic and Macromolecular Chemistry, Ghent University, Krijgslaan 281-S4, 9000 Ghent, Belgium
[2]Center for Advanced Preclinical Imaging (CAPI), First Faculty of Medicine, Charles University, Salmovská 3, 120 00 Prague 2, Czech Republic
[3]Institute of Organic Chemistry and Biochemistry, Czech Academy of Sciences, Flemingovo sq. 2, Prague 6, 160 00
[4]Institute of Biophysics and Informatics, Charles University, First Faculty of Medicine, Salmovská 1, 12000 Prague 2, Czech Republic
[5]BIO INX, Technologiepark-Zwijnaarde 66, 9052 Ghent, Belgium
[6]4Tissue, Technologiepark-Zwijnaarde 48, 9052 Ghent, Belgium

[*]Corresponding author, email: Sandra.VanVlierberghe@UGent.be, Kristyna.Kolouchova@UGent.be

Keywords: tissue engineering, photo-crosslinkable gelatin, fluorine-19 magnetic resonance imaging, poly[*N*-(2,2-difluorethylacrylamide)], digital light processing

Mechanical evaluation of the gel-MA-AEMA-F hydrogels via oscillatory rheology

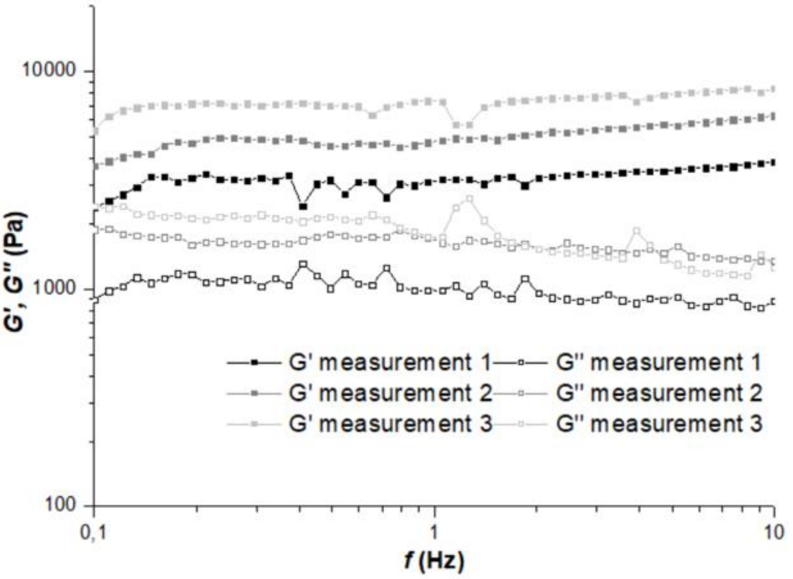

Figure S1. Mechanical spectrum of the hydrogel **gel-MA-AEMA** (15 w/v %, 2 mol. % of PI resp. to MA/AEMA content). The storage (*G'*) and loss (*G''*) moduli were measured at 37 °C, normal force 0.6 N, and strain of 0.1%. The experiment was measured in three runs.

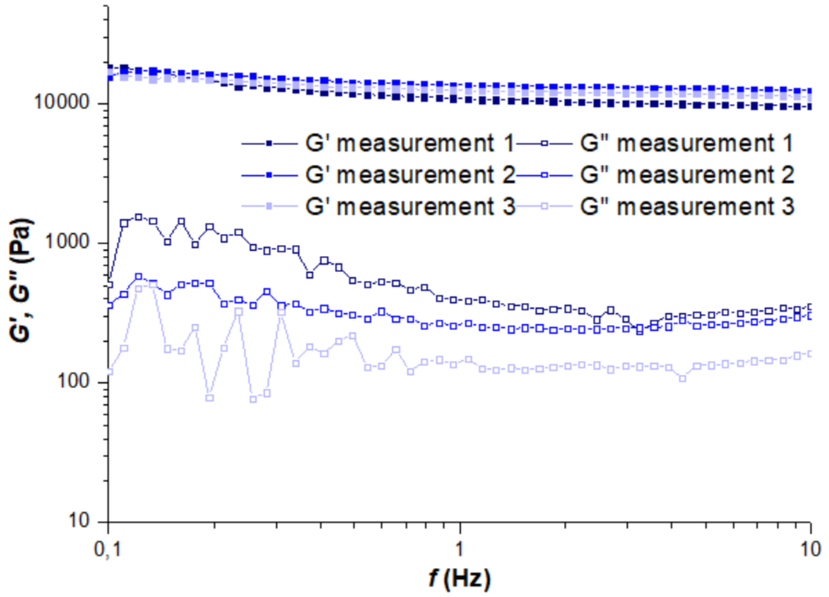

Figure S2. Mechanical spectrum of the hydrogel **gel-MA-AEMA-F** (2 mol. % of PI resp. to MA/AEMA content). The storage (*G'*) and loss (*G''*) moduli were measured at 37 °C, normal force 0.6 N, and strain of 0.1%. The experiment was measured in three runs.

Biomaterial ink development

Table S1. The effect of content of Li-TPO-L (photo-initiator) and tartrazine (photo-absorber) in the starting solution for DLP optimization on its t_g measured by rheology.

Conditions	1	2	3	4	5
**Li-TPO-L (mol. %)**	10.0	15.0	20.0	20.0	20.0
**PA (mol. %)**	0.0	0.0	0.0	0.50	0.80
**t_g ± SD (s)**	3.2 ± 0.3	2.8 ± 0.3	2.5 ± 0.0	5.3 ± 1.9	7.8 ± 1.2

*Corresponding to mol. % of MA/AEMA in the solution. SD calculated for n = 3.

Table S1 concludes the obtained t_{gel} values as a function of PI (Li-TPO-L [53,54]) and PA (tartrazine[55–57]) concentration utilized for each biomaterial ink formulation, measured at 400 < λ < 500 nm, I = 19.51 mW/cm^2. Various Li-TPO-L concentrations between 10-20 mol% with regards to the amount of crosslinkable groups present in the hydrogel precursors were tested. Upon increasing the Li-TPO-L from 10 mol% to 20 mol% in the biomaterial ink formulations, t_{gel} decreased to 2.5 ± 0.0 s and this concentration was utilized for the further formulations to enable reduced crosslinking times of the hydrogel precursors.

Tartrazine was used as photoabsorber, since it offers biocompatibility and yet outstanding absorbance in the visible light range (λ = 310–530 nm, with a λ_{max} = 425 nm) [55–57] overlapping with the wavelength of the DLP 3D printer (λ = 405 nm). Upon supplementing the biomaterial ink with 0.5-0.8 mol% tartrazine, the t_{gel} increased (from 2.5 ± 0.0 s up to 7.8 ± 1.2 s). The aim was to maintain the t_{gel} as low as possible to obtain time-efficient crosslinking during additive manufacturing yet obtain a sufficient CAD/CAM mimicry. Therefore, the determination of the final PA amount was assessed during DLP additive manufacturing whilst evaluating the post-printing scaffold parameters. The final PA concentration utilized in the system was chosen as of 0.72 mol%.

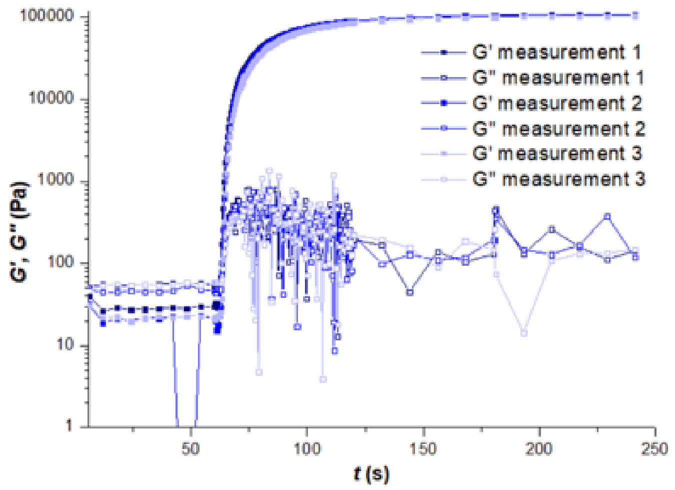

Figure S3. Evolution of the storage modulus of 15 *wt.* % **gel-MA-AEMA-F** in DMSO with the 10 mol. % concentration of PI, respectively to molar amount of the MA/AEMA, during UV-A-induced cross-linking at 37 °C as determined by rheology. The experiment was measured in three runs.

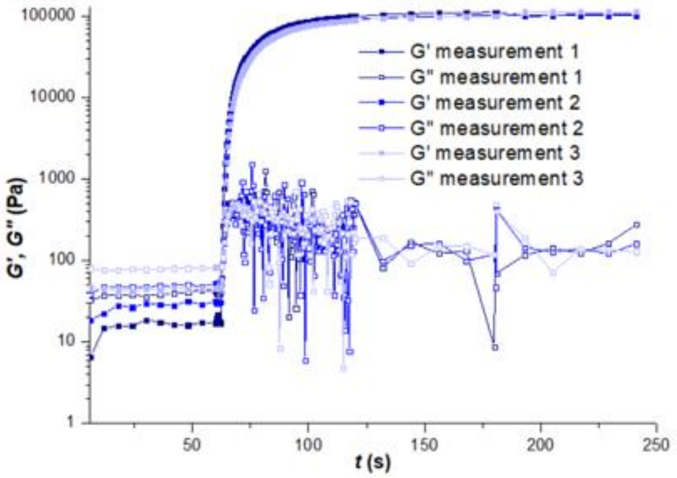

Figure S4. Evolution of the storage modulus of 15 *wt.* % **gel-MA-AEMA-F** in DMSO with the 15 mol. % concentration of PI, respectively to molar amount of the MA/AEMA, during UV-A-induced cross-linking at 37 °C as determined by rheology. The experiment was measured in three runs.

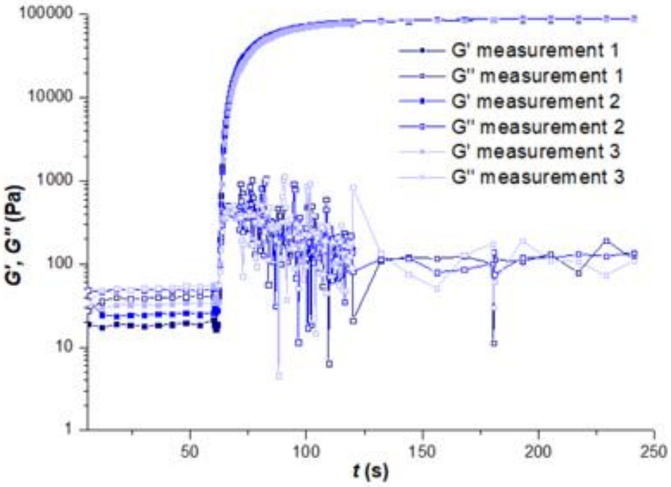

Figure S5. Evolution of the storage modulus of 15 *wt*. % **gel-MA-AEMA-F** in DMSO with the 20 mol. % concentration of PI, respectively to molar amount of the MA/AEMA, during UV-A-induced cross-linking at 37 °C as determined by rheology. The experiment was measured in three runs.

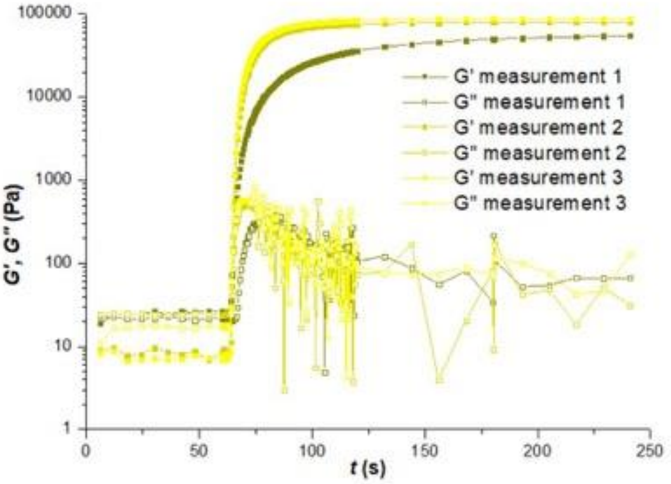

Figure S6. Evolution of the storage modulus of 15 *wt*. % **gel-MA-AEMA-F** in DMSO with the 20 mol. % concentration of PI and 0.5 mol. % concentration of PA, respectively to molar amount of the MA/AEMA, during UV-A-induced cross-linking at 37 °C as determined by rheology. The experiment was measured in three runs.

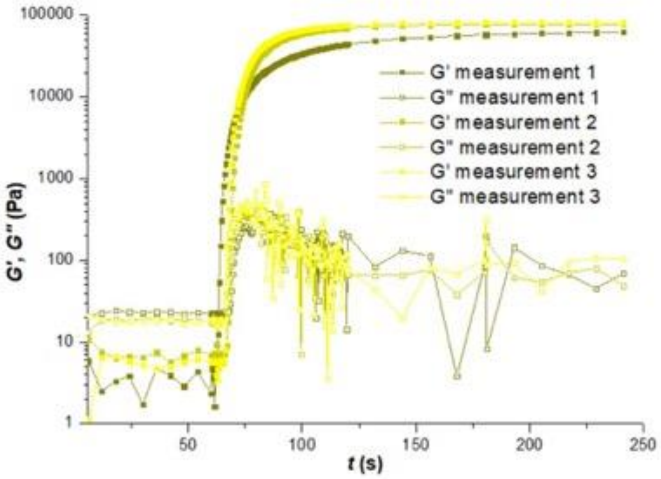

Figure S7. Evolution of the storage modulus of 15 *wt*. % **gel-MA-AEMA-F** in DMSO with the 20 mol. % concentration of PI and 0.8 mol. % concentration of PA, respectively to molar amount of the MA/AEMA, during UV-A-induced cross-linking at 37 °C as determined by rheology. The experiment was measured in three runs.

High resolution magic angle spinning (HR-MAS) spectroscopy

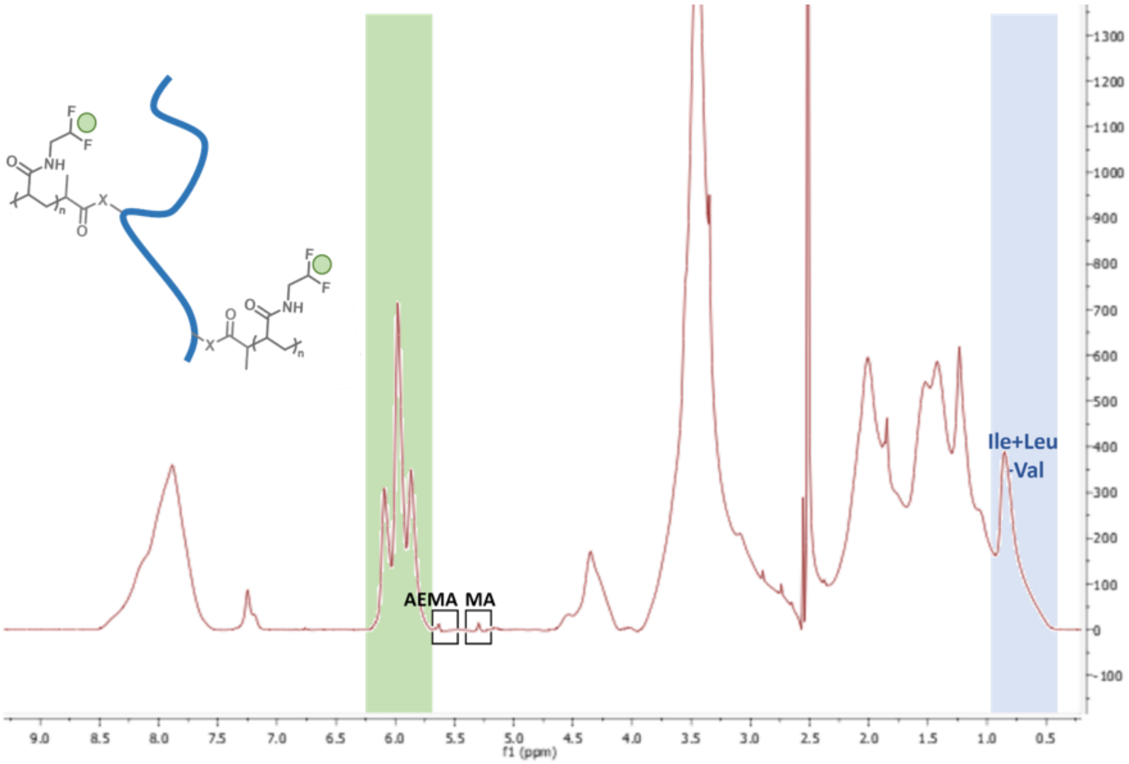

Figure S8. ^{1}H HR MAS NMR of a sample **gel-MA-AEMA-F** swollen in DMSO-*d6* measured using Bruker Avance 400 MHz spectrometer (9.4 T wide bore magnet) equipped with a 4 mm BL4 X/Y/H probe. Magic angle spinning was performed at 4 kHz using ceramic zirconia rotors of 4 mm in diameter. Acquisition parameters used were the following: a spectral width of 8 kHz, a 90° pulse length of 3.5 μs, an acquisition time of 1.7 s, a recycle delay time of 15 s and about 64 accumulations.

Biological evaluation of the gel-MA-AEMA-F hydrogel scaffolds

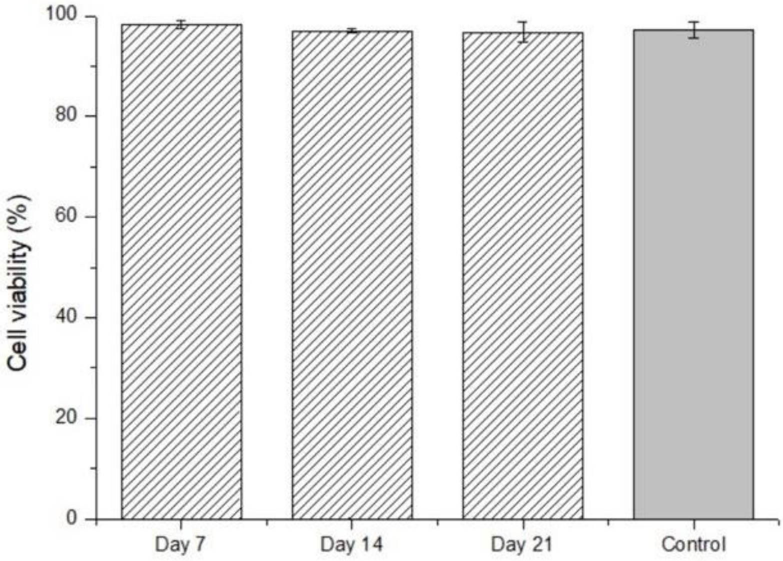

Figure S9. Evaluation of *in vitro* live/dead assay of seeded adipose tissue-derived stem cells onto scaffolds after 7, 14, and 21 d compared to the control (osteogenic tissue-derived stem cells seeded on a well plate), evaluated after 7 d.

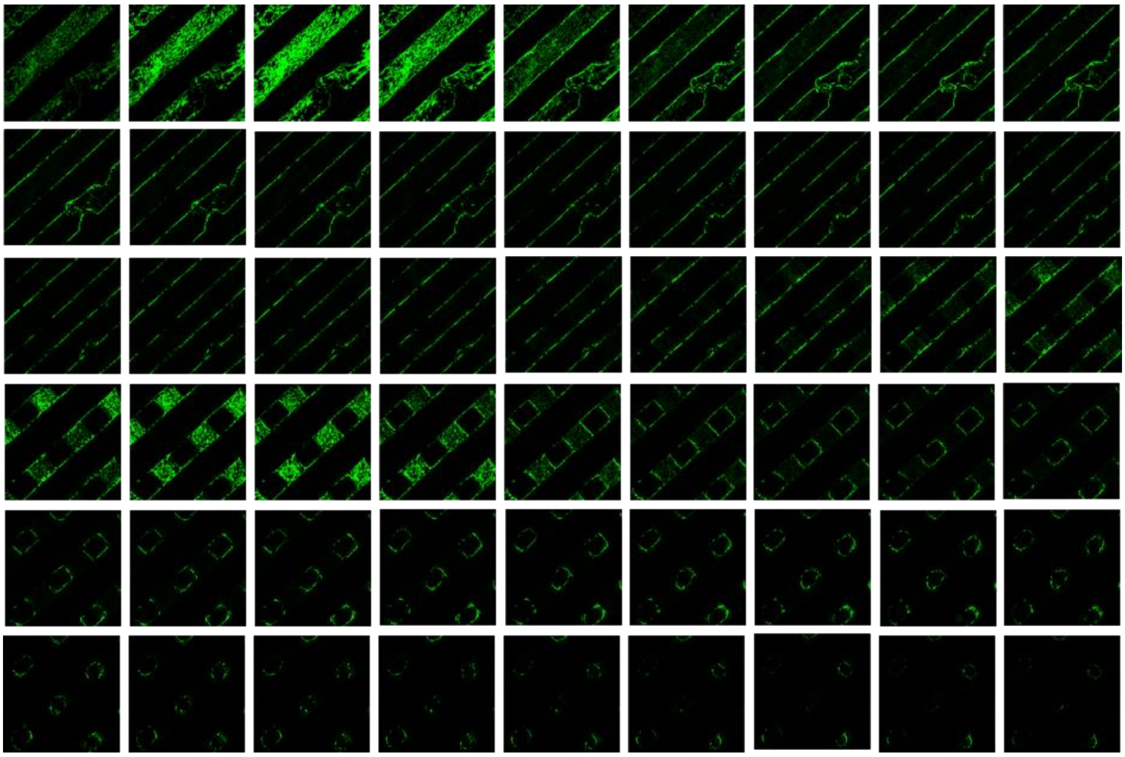

Figure S10. *In vitro* live/dead staining of seeded adipose tissue-derived stem cells after 7 d (1 selected scaffold from 3). Series of stack images (2 µm thick) of populated scaffold.

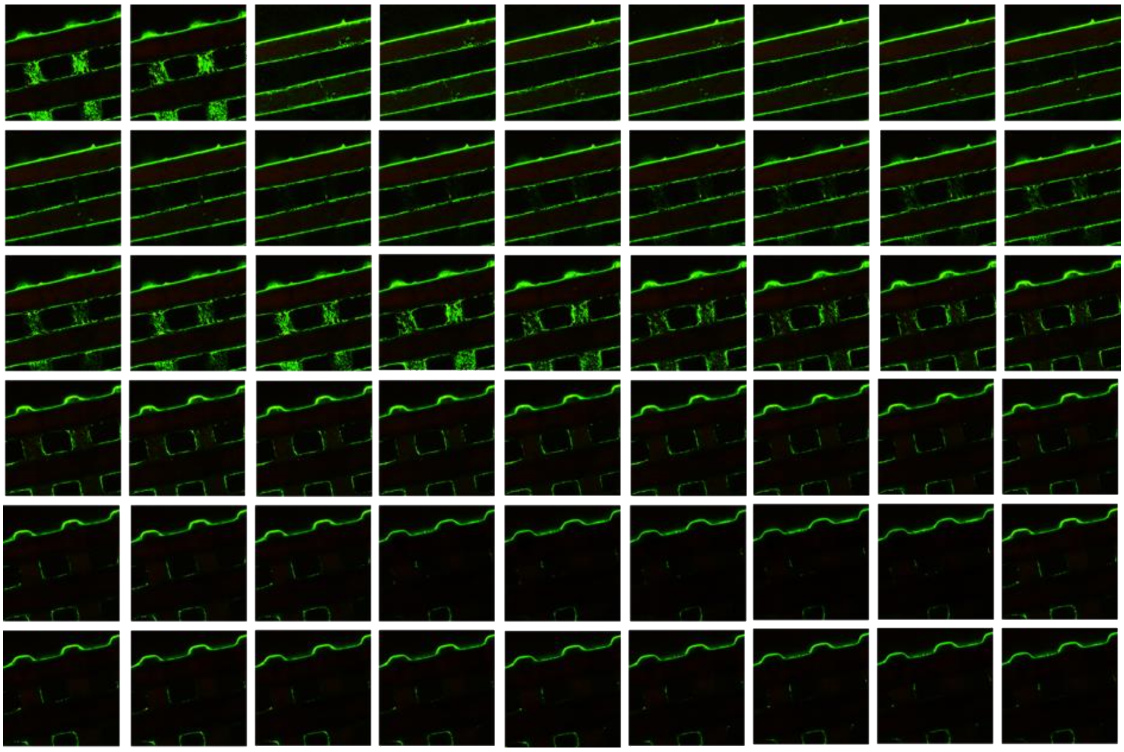

Figure S11. *In vitro* live/dead staining of seeded adipose tissue-derived stem cells after 14 d (1 selected scaffold from 3). Series of stack images (2 µm thick) of populated scaffold.

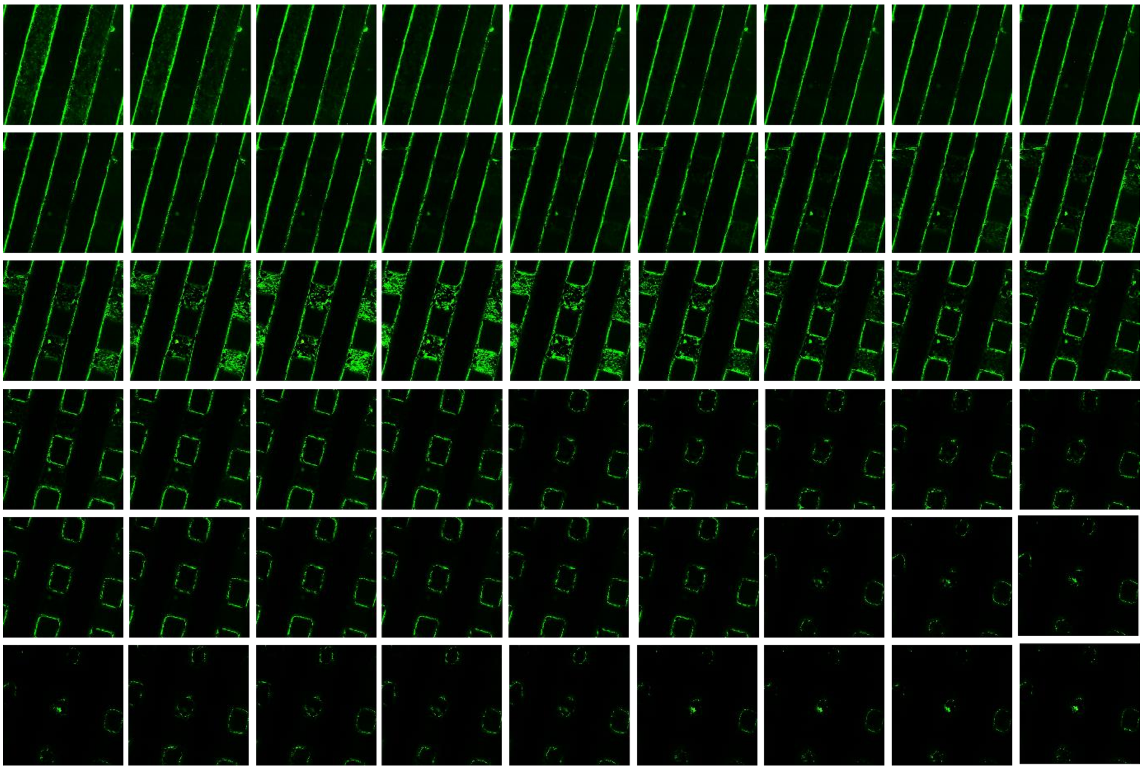

Figure S12. *In vitro* live/dead staining of seeded adipose tissue-derived stem cells after 21 d (1 selected scaffold from 3). Series of stack images (2 µm thick) of populated scaffold.

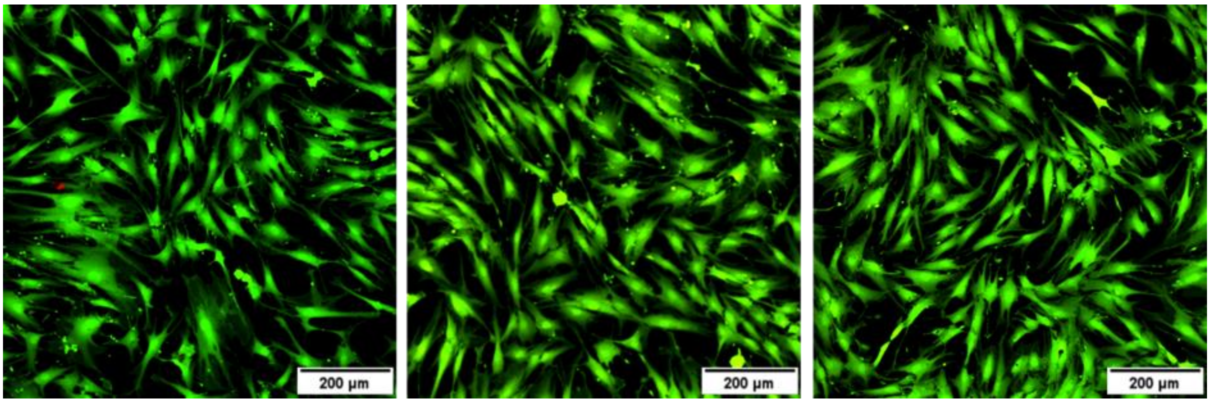

Figure S13. *In vitro* live/dead staining of 3 control samples, the adipose tissue-derived stem cells seeded on a well plate, after 7 d.

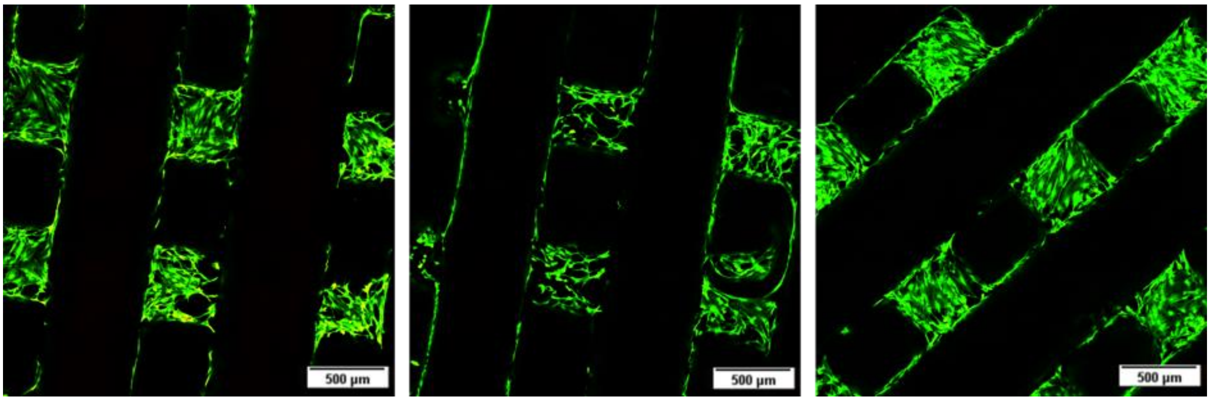

Figure S14. *In vitro* live/dead staining of adipose tissue-derived stem cells seeded on 3 printed scaffolds after 7 d.

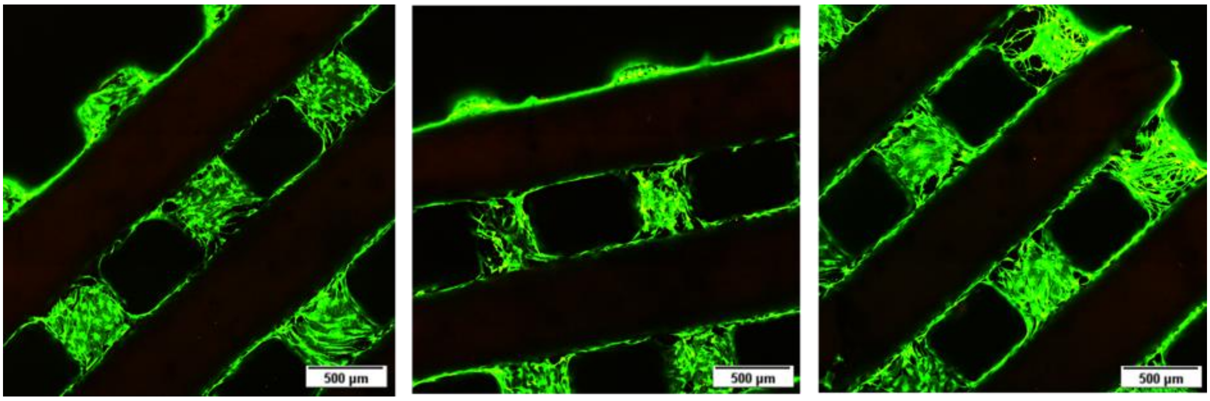

Figure S15. *In vitro* live/dead staining of adipose tissue-derived stem cells seeded on 3 printed scaffolds after 14 d.

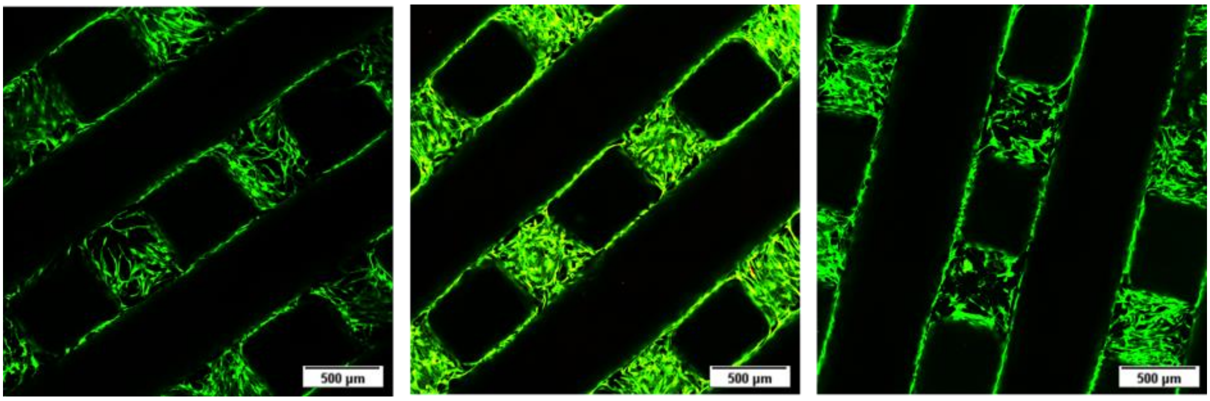

Figure S16. *In vitro* live/dead staining of adipose tissue-derived stem cells seeded on 3 printed scaffolds after 21 d.

References

[1] C. Ma, W. Li, D. Li, M. Chen, M. Wang, L. Jiang, L. S. Mille, C. E. Garciamendez, Z. Zhao, Q. Zhou, Y. S. Zhang, J. Yao, *Biofabrication* **2022**, *14*, DOI 10.1088/1758-5090/ac49d5.

[2] M. Wang, W. Li, J. Hao, A. Gonzales, Z. Zhao, R. S. Flores, X. Kuang, X. Mu, T. Ching, G. Tang, Z. Luo, C. E. Garciamendez-Mijares, J. K. Sahoo, M. F. Wells, G. Niu, P. Agrawal, A. Quiñones-Hinojosa, K. Eggan, Y. S. Zhang, *Nat Commun* **2022**, *13*, DOI 10.1038/s41467-022-31002-2.

[3] M. Leulescu, A. Rotaru, I. Pălărie, A. Moanţă, N. Cioateră, M. Popescu, E. Morîntale, M. V. Bubulică, G. Florian, A. Hărăbor, P. Rotaru, *J Therm Anal Calorim* **2018**, *134*, 209.

[4] B. Grigoryan, S. J. Paulsen, D. C. Corbett, D. W. Sazer, C. L. Fortin, A. J. Zaita, P. T. Greenfield, N. J. Calafat, J. P. Gounley, A. H. Ta, F. Johansson, A. Randles, J. E. Rosenkrantz, J. D. Louis-Rosenberg, P. A. Galie, K. R. Stevens, J. S. Miller, *Science (1979)* **2019**, *364*, 458.

[5] H. Gong, B. P. Bickham, A. T. Woolley, G. P. Nordin, *Lab Chip* **2017**, *17*, 2899.
